# Irish Roma: a literature review

**DOI:** 10.1007/s11845-022-03054-2

**Published:** 2022-06-18

**Authors:** Aoife O’Sullivan, Darragh Rooney, Clodagh S. O’Gorman, Anne Marie Murphy

**Affiliations:** 1 Department of Paediatrics, University Hospital Limerick (UHL), Limerick, Ireland; 2 Department of Paediatrics, School of Medicine, University of Limerick (UL), Limerick, Ireland

**Keywords:** Children of Roma, Cultural competence, Health inequality, Ireland’s nomadic races, Irish Roma, Traveller separateness

## Abstract

It is estimated that the Roma are the largest ethnic minority population in Europe (HSE in Roma Intercultural Guide, 2020). There is a dearth of information in the Irish medical literature on the Roma in Ireland. The aim of this paper is to provide an overview of the Roma in Ireland, to identify Roma-specific culture, family structure, paediatric illness, and health equality within the context of the Irish population. To do this, a review was completed of the English language literature on Roma available from 2010 to 2021 using web of science databases. Relevant clinicians and organisations were contacted to compile data on the Irish Roma to inform appropriate action in Roma child health. Up until 2021, the national census in Ireland did not include Roma as a category in ethnicity (HSE in Roma Intercultural Guide, 2020). As such, it is difficult to get an accurate number of the population in Ireland. Pavee Point Traveller and Roma Centre in 2009 estimated a population of approximately 5000 (National Traveller and Roma Inclusion Strategy in Justice.ie, 2017). The majority of the Roma in Ireland are Romanian (National Traveller and Roma Inclusion Strategy in Justice.ie, 2017). There is limited understanding of their culture in Ireland (National Traveller and Roma Inclusion Strategy in Justice.ie, 2017). Often overlooked, small indigenous groups or nomadic races have unmet medical needs (National Traveller and Roma Inclusion Strategy in Justice.ie, 2017). Across Europe, they have a lower life expectancy and higher burden of illness due to lower socioeconomic status, discrimination, and poor access to health services (National Traveller and Roma Inclusion Strategy in Justice.ie, 2017). Cultural competence is necessary to provide effective healthcare.

## Introduction

The European Union (EU) uses the term “Roma” to describe several minority groups with diverse cultural backgrounds including Roma, Gypsies, Sinti, and Travellers [2, 3]. For this review, the term “Roma” is specific to the Roma population and “traveller” refers to Irish travellers.

According to the HSE intercultural guide, it is estimated that the Roma population is the largest ethnic minority group in Europe [1]. They began migrating to Europe from Northeast India from 1000 A.D. [1]. Approximately six million live throughout the EU [1]. Statistically, the largest Roma communities live in Romania, Bulgaria, Hungary, and Slovak Republic [1]. The majority of Roma in Ireland are from Romania and Slovak Republic. In general, Roma in Ireland are EU citizens, and many are Irish citizens [2]. The title “Roma” is an umbrella term due to sub-groups within the Roma population with differences in cultural tradition and language [2]. The Roma Chakra is the official symbol adopted by the Roma in 1971 [1]. It encompasses Roma Indian heritage and represents the vardo/wagon which was the traditional home of the Roma for over 100 years [1].

**Figure:**
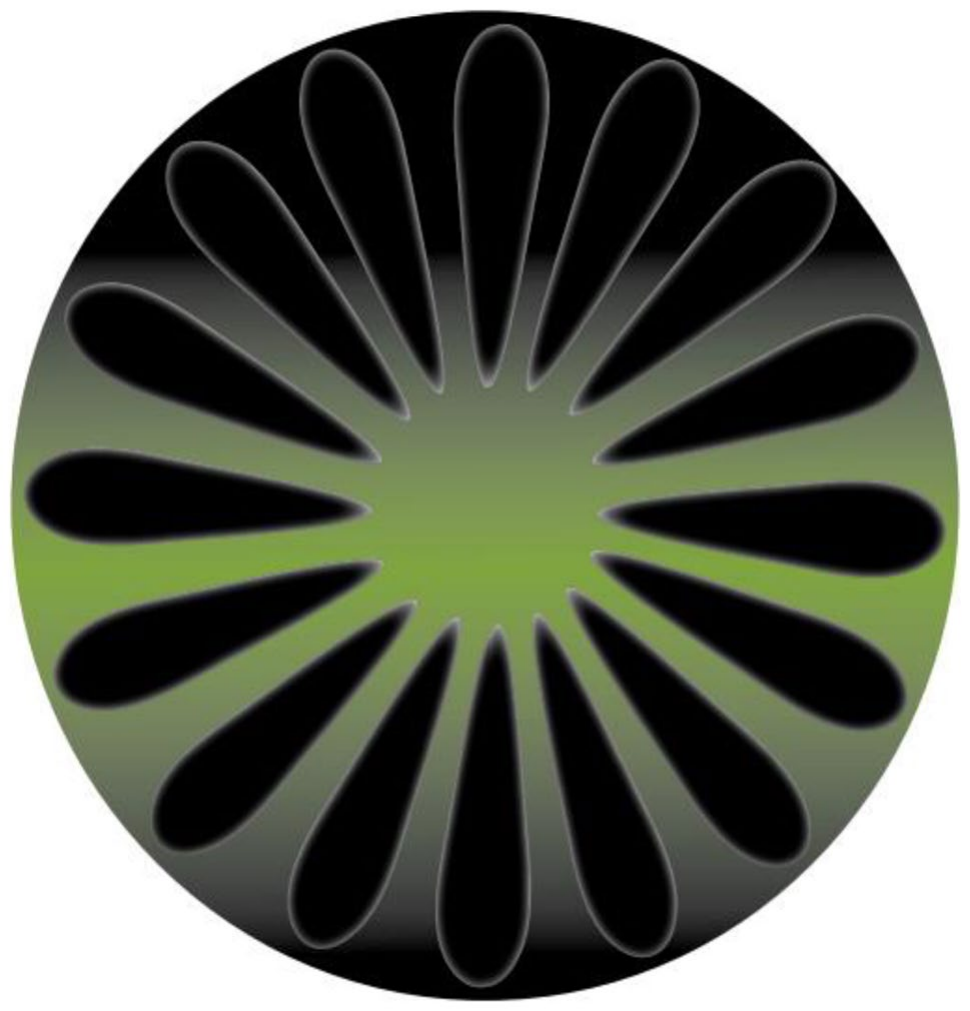
The Roma Chakra. HSE, Roma Intercultural Guide (2020) [1]

In 2014, the World Health Organisation estimated that Roma has one of the largest populations younger than 16 years and a very low percentage older than 65 years [2, 3]. This demonstrates their high birth rate and premature death rate [2, 3]. Roma women tend to marry young, often less than 20 years [2]. Traditionally, their culture was patriarchal with male dominance [2]. Roma women are now more progressive, and some participate in education and paid work [2].

In Ireland, evidence suggests most of the Roma population identify as Pentecostal [1]. But there are also Orthodox and Roman Catholic. Traditional Roma female dress includes a colourful blouse, long skirt, scarf, and jewellery [1]. However, many Roma no longer identify by their dress [1, 2]. The importance of the Roma extended family and the Romani language are unique to Roma culture [1, 2]. The family unit is core to Roma culture; in some cases, there may be three generations of Roma living under one roof [1, 2]. This is important for hospital visitation, medical appointments, and accurate booking of interpreters, including use of family members and the HSE emergency multilingual aid in acute scenarios [1]. Romani is the official language of the Roma with different dialects of the language across sub-groups and regions [2]. Traditionally, age hierarchy existed amongst Roma, with older family members responsible for important decisions including healthcare [1, 2]. It is important for health service providers to be accustomed to the advantages and disadvantages of this custom; there is often a collective family response to serious illness with dedicated support networks [1, 2].

## Results

### Irish Roma and children of Roma

Prior to the development of the EU, Roma emigrated to Ireland as short-term workers mostly for farm labour, others sought asylum [2]. It is estimated from the 2018 National Roma Needs Assessment (NRNA) and Pavee Point that there are approximately 5000 Roma living in Ireland [2, 3]. This estimate does not interpret to which Roma sub-groups these some 5000 Roma belong [2]. Traditionally described as a nomadic group, the Irish Roma today are generally in semi-fixed/ fixed accommodation [3], although statistically there is a high rate of homelessness and overcrowded accommodation [3–5]. In the EU, approximately 80% of Roma are at risk of poverty, in comparison to 17% for non-Roma [2]. They maintain their distinct identity through dress, close extended family, health, and social cultural beliefs [3]. This unique identity can highlight the differences in culture with the majority population and contribute to marginalisation [4–6]. Evidence suggests poor engagement in Irish society attributable to marginalisation, discrimination, poor access to health and social services, and lack of a political voice [4].

In 2014, the Ombudsman for Children published the “Logan Report” in relation to the inappropriate removal of two Roma children from their families under Sect. 12 of Childcare Act 1991 [6]. This included child A from Athlone and child T from Tallaght [6]. Both children were reported as potential abductions by members of the public to the Gardai based on their fair complexions in comparison to that of their families [6]. Both children in question suffered from oculocutaneous albinism [6]. It is important to note that at this time, there was an international media storm surrounding the case “Maria” in Greece, a young girl who was abducted by a Roma family [6].

The “Logan” report looked at the circumstances surrounding this error and the relationship between public services and the Roma community [6]. All recommendations from this report were accepted by the state [6]. Recommendations included an apology from the Minister for Justice and Equality to the families involved, provision of healthcare support physical and psychological for both children, improved access and availability of Roma cultural mediators and interpreters, accurate reporting on Roma by media outlets in relation to social problems and crime, and an assessment of the needs of the Irish Roma community. This assessment of need was published as the NRNA in 2018 [6].

The NRNA included Roma researchers at all stages [5]. It was both quantitative with questionnaires and qualitative with focus groups and interviews with Roma across Ireland [5]. Males and females took part with most participants aged 18–34 years [5]. All participants over 18 were born outside Ireland, but 63.3% of minors were born in Ireland [5]. This demonstrated that there are 2nd- and 3rd-generation Roma living in Ireland, indicating Roma have been part of Irish society for some time [5]. But only approximately 5% of adult Irish Roma had Irish citizenship [5]. Findings suggested that 20% of Roma had no social welfare support including child benefit, no housing support including homeless support, and no employment support [5]. Many of these cases were due to failure to supply proof of habitual residence including a PPS (Personal Public Services Number) [5]. For a PPS, proof of identity e.g. valid passport, proof of address, and a valid reason for requiring a PPS is required [5]. Others feared engaging with the state due to previous negative experience in their country of origin [5].

Many Roma are unable to meet the criteria for habitual residence which considers main centre of interest, length and continuity of presence, length and reason for absence, and future intention [5]. If they do not satisfy the habitual residence conditions, they and their family are not entitled to social protection including income, healthcare, accommodation, and disability support [7]. The NRNA found poverty amongst Roma to be a core issue, preventing Roma from participating fully in Irish life [5]. The NRNA was completed and published by Pavee Point [5]. Pavee Point is a voluntary non-governmental organisation (NGO) that acts as an advocate for Roma but also traveller rights in Ireland [5]. It has had a Roma project since 2000; this was set-up to assist Roma in accessing services, participating in Irish society, and preventing discrimination [5].

A smaller Roma study was completed in 2016 locally in Balbriggan by Musicantia, a Roma support centre set-up in 2013 [7]. The study was completed over a year, which hired Roma researchers, and the method was a questionnaire [7]. Thirty Roma participated similarly to the NRNA, majority are female, and the average age of participants was approximately 35 years [7]. The average number of children per family was four, three times the national average in 2016 [7]. Thirteen percent of respondents were married and 77% had children, suggesting a high number of single parents [7]. As for NRNA, the most common religion was Pentecostal, and the most common first language was Romani [5, 7]. Most participants had sought asylum in Ireland prior to 2007 when Romania joined the EU; all were resident in Ireland for greater than five years and 63% for greater than ten years [7]. In relation to health, 97% had a medical card, but 77% found language a barrier to accessing healthcare, of note 46% of respondents had no English or beginners level [7]. 64% had initially found it difficult to acquire housing, 97% were in private rented accommodation, and none were homeowners [7]. There was a low level of education, with average time in school of five years amongst participants [7]. However, most of their children were enrolled in school in Balbriggan and had integrated well [7]. 90% were unemployed and in receipt of social welfare, although respondents highlighted the difficulties in meeting the habitual residence condition as per NRNA [5, 7]. The two main recommendations from this study were in relation to racism and low English literacy levels [7]. 79% state they have been the target of racism or discrimination; reports included services such as healthcare, schools, workspaces, and dealings with the Gardai [7]. The recommendations sought provision of English language classes for Roma communities, and expansion of anti-racism initiatives, e.g. “yellow flag programme in schools” [7].

The EU commission describes lack of trust between Roma and mainstream society [8]. The EU Framework for National Traveller and Roma Integration Strategies to 2020 was to promote social and economic inclusion [2]. As part of this in Ireland, the National Traveller and Roma Inclusion Strategy (NTRIS) 2017–2021 was developed [2, 5, 8]. It focuses on health, housing, education, and employment [2, 5].

In relation to health, Roma should have improved access and participation within Irish healthcare [2]. This includes consideration given to needs of Roma with mental health and substance abuse issues, representation on local and national health structures, improved treatment of chronic conditions, improved access for maternal health, establish ethnic identifier to monitor use of services, provision of medical cards to Roma with no income, culturally appropriate service delivery, and secure funding specific to Roma health [2]. For housing provision, it should be culturally appropriate and monitored [2]. Outcomes in relation to education should be equal to population majority [2]. This includes facilitating access to the free pre-school scheme under early childcare and education up to age 3 years, early intervention to promote retention to leaving certificate level, support parental involvement, 3rd level peer mentorship programme, transparency, and fairness for admission to all levels of education and educator inclusion training and guidelines [2]. For employment, the strategy was to increase opportunities for Roma including apprenticeship and training schemes [2].

### Cultural competence

Cultural differences can be associated with racial connotations such as thieves, criminals, and beggars [8]. Discrimination makes it difficult to have a voice politically and socially [8]. The EU commission suggests that discrimination starts at an early age [8]. Across member states, Roma children often attend segregated schools or classes, 58% in Slovakia and 45% in Hungary [8]. Approximately 54% of Roma suffer discrimination when seeking paid work [8].

The Roma have experienced a long history of exclusion, including slavery and the holocaust [5]. In Bulgaria in the late 1950s, Roma children were forbidden to speak Romani in school and had to adopt a Slavic name [5]. In Czechoslovakia, in the 1970s/1980s, Roma women were sterilized to reduce reproduction rate to prevent “social risk” posed by Roma [5].

During the expansion of the EU from 2004, there was an increase in migration from Eastern to Western Europe [5]. The Irish media portrayed a negative image of “fear” surrounding migration of Roma, including lack of contribution to society and abuse of social welfare [5]. Statistics suggest that the reason for emigration of Roma was like that of non-Roma, employment, better housing, and better education [5]. Poor data collection and absence of a Roma category under ethnicity in the Irish census makes it difficult to determine the true socioeconomic status of Irish Roma [1, 5].

In 2014, the Logan report addressed issues relating to cultural competence within the public sector [6]. Addressing this report, in the same year, an e-learning course for frontline public servants was developed, “delivering equality in public services” [9]. Other improvements following this report included training for ethnic liaison officers within an Garda Siochana, intercultural guidelines for primary schools, Education (admissions to school) Bill 2014 for fair admission to school, and the Roma health project Tallaght [9].

In 2015, a report by the United Nations described Europe as collectively discriminatory towards Roma [5]. It noted that media outlets focussed only on social problems and crime when reporting on Roma [5]. In 2014, the Irish times reported that approximately four Roma families in Waterford were forced to leave their homes due to racist abuse [5]. In the same year, the Irish immigrant support centre rolled out a pilot anti-racism training programme to 20 Gardai in Cork [10]. It involved promoting awareness of the impact racism has on minority populations and how to prevent discriminatory ethnic profiling [10].

The NRNA recommended monitoring of socioeconomic status of Irish Roma using an NGO that works with Roma on the ground for accurate data [5]. It recommended improving access to support for Roma with birth registration, citizenship, and social welfare applications [5]. In this report, Roma described discrimination relating to traditional dress, skin colour, or just looking “non-Irish” [5]. Roma women, those who are illiterate, non-English speakers were considered most vulnerable to anti-Roma abuse [10]. The NRNA highlighted the importance of interpreters for improved service access [5]. In relation to housing, some landlords openly stated they did not accept Roma tenants [5]. A total of 78.9% of Roma who responded to a NRNA questionnaire reported discrimination when seeking work and often hid their identity [5]. Racism was also reported in relation to health services, public spaces including restaurants, and interactions with Gardai [5].

### Traveller separateness

Like other minority groups, Irish travellers and Roma have their own language, traditions, and customs [11]. They also have similarities including nomadism and large close family networks [12]. This includes shared experiences of racism and discrimination based on their ethnicity [12].

Unlike the Roma, Irish travellers have been a minority group in Ireland for centuries [12]. According to the 2016 census, the traveller population was greater than six times that of the Roma population at approximately 0.7% of the total population, 30,987 [13]. It is important to note that the All-Ireland Traveller Health Study in 2010 estimated the traveller population at greater than 36,000 [13]. It is likely that both Roma and traveller populations are underestimated due to lack of accurate data [13]. For the NTRIS, most data in terms of population statistics for the traveller and Roma communities came from the All-Ireland Traveller Health Study in 2010 and the NRNA 2018 [2, 5, 9, 14]. Following recommendations from NTRIS, Roma will be included in the ethnicity section of census 2021 [2, 13].

In terms of Irish legislation, the Prohibition of Incitement to Hatred Act 1989, the Unfair Dismissals Acts 1977, the Employment Equality Acts, and the Equal Status Acts refer specifically to travellers as a protected group [13]. Unlike Roma, travellers are referred to by name within Irish legislation in relation to protections of ethnic minorities under EU directives [9]. Both Roma and travellers are named under the Social Inclusion and Community Activation Programme (SICAP) developed in 2015 [9]. SICAP through engagement with the community and local organisations such as Pavee Point aims to target those most vulnerable to social exclusion and poor access to education and training [9].

The Irish traveller community has several government and NGO working on their behalf including Pavee Point, local traveller inter-agency groups, e.g., Donegal traveller project, and community traveller health units [9]. The national traveller accommodation consultative committee was established initially in 1999 [9]. It includes construction works, house acquisition, and provision of temporary accommodation [9]. No such committee exists for the Roma population [9]. Similarly in relation to healthcare, the national traveller health advisory forum includes HSE staff, traveller representatives, and traveller health units [9]. Traveller health units run projects such as asthma education for travellers [9]. This was developed due to high rates of asthma in the national All Ireland Traveller Health Study [9, 14]. In 2010, asthma accounted for 71.9% of reported chronic illness in traveller children [14]. It is noteworthy that 83% of travellers surveyed in the 2010 health study sought and received healthcare advice from traveller-specific projects and organisations [14]. It may be hypothesized from this data that the Roma population may benefit from similar Roma-specific health initiatives. Both travellers and Roma have the same rights as any other EU citizen living in Ireland, but as a Roma, there are few public-led projects targeted specifically to them and their diverse cultural needs [2, 5, 9]. One example of a health-directed project specific to Roma is the Tallaght Roma Integration Project (TRIP) [15].

### Health inequality

Studies suggest that Roma people may mistrust health services due to discrimination, cost, cultural barriers, language, and difficulty accessing care [3, 9, 16]. In the UK, it has been found that Roma has a lower life expectancy and poorer health outcomes than the general population [17, 18]. The 2014 Roma health report by the European Commission found Roma life expectancy to be 10–15 years less than the general population [16]. Roma in the UK and in Europe are associated with higher maternal and infant mortality, higher rates of paediatric emergency department attendances, accidental injury, and infections [3, 17]. They also have lower rates of paediatric immunisation and general practice visits [17]. Roma infants are more prone to malnutrition, diabetes, and poor oral hygiene [16, 18]. There is a higher prevalence of inherited rare disorders amongst Roma children due to practice of consanguineous marriage in some clans. There is a need for culturally appropriate genetic counselling [16]. There is also less uptake of preventative health services by Roma adults [16, 18]. They have higher rates of chronic illness including coronary artery disease, infectious disease, mental illness, and substance abuse [15, 16].

Mistrust of health services may lead to lower utilisation and poorer health outcomes, particularly if they have experienced segregation and racism in their country of origin [3, 10, 15, 16, 18]. Roma community engagement in the planning and delivery of health services may improve trust and utilisation [3, 15]. Access to healthcare is particularly difficult for Roma whose status in Ireland is unresolved [15]. In 2009, the TRIP was developed as a community primary care model targeting Roma [15]. It is run by the SafetyNet primary care network in Tallaght [15]. The service offers acute and chronic illness management, ante-natal care, and paediatric services [15]. Following a community consultation process with Roma in Tallaght through use of inter-agency groups and Roma cultural mediators, one of its main goals is to support Roma in accessing healthcare services [15]. Inability to access health services was directly related to an inability to pay healthcare costs and/ or ineligibility for social welfare [15].

Tallaght has a relatively large migrant population with approximately 25% of the population in west Tallaght from a migrant background including the Roma community [15]. The TRIP estimates the Roma population in Tallaght to be between 600 and 1000, but it is important to note that the ethnic identity of service users is not formally documented [15]. In 2012, the TRIP initiated the pilot Roma primary care initiative under SafetyNet [15]. This involved a mobile GP clinic set-up in the carpark of Tallaght hospital providing free primary care and support services including interpreters for Roma [15]. Due to high demand, this service has become established as part of Tallaght primary care centre, and no longer takes place in the car park [15]. In 2014, 892 Roma had availed of this service, with 70% female service users [15]. The high rate of female service use prompted development of women’s health initiatives including reproductive health and contraception [15]. In relation to children’s health, 159 children less than 5 years old attended in 2014 [15]. As a result, a vaccination programme has been developed as part of the service [15]. This is important as across Europe low uptake of childhood immunisation is a concern amongst Roma [16]. It is suggested that this is due to vaccination myths, low levels of education, illiteracy, and fear of public service use [16].

Another key issue for Roma was access to maternal health services [15, 16]. The NRNA found that for 24% of pregnant Roma women, their first interaction with maternal health services was when giving birth [5]. There is limited data on the health of Romani women in Europe [16]. The highest birth rate has been found in the most socially disadvantaged Roma families [16]. Rates of teenage pregnancy are higher amongst Roma than non-Roma in Europe [16]. According to the 2014 Roma health report, the average age was 17.27 years in the 2011 survey [16]. This is important from an Irish context as under Sect. 5 of the Criminal Law (Sexual Offences) (Amendment) Act 2007 the age of consent for sexual relations is 17 years [19]. The EU recognises the high birth rates amongst Roma women [16]. This may be due to cultural beliefs; traditionally, Roma men valued women by the number of children they gave birth to [16]. A nullipara female is considered shameful for her and her extended family [16]. Traditionally, birth must take place outside the home, as it is seen as impure [16]. Teenage pregnancy and lack of utilisation of prenatal obstetric services puts Roma women and children at increased risk of complications. The 2014 Roma health report found that in Hungary, Czech Republic, and Slovakia, there are higher rates of abortion, infant mortality, low birth weights, and premature births [16]. Additionally, Roma women less frequently visit a Gynaecologist than the general population in Europe and have lower uptake of cervical screening programmes [16].

The Decade of Roma Inclusion (DRI) between 2005 and 2015 brought several European governments together to tackle discrimination against the Roma, including health inequality [2, 8]. Discussions involved members of the Roma community and focused on the integration of Roma into European society [2, 8]. It was felt by members of the DRI that there were four main priorities to alleviate health inequality [2, 8]: culturally sensitive health policies, greater sensitivity given to the values and needs of the Roma community, involvement of Roma as key stakeholders in policy making, and legitimization of the Roma population [2, 8].

The NTRIS has set out recommendations for health equality including (a) extended health and basic social security coverage and services; (b) awareness campaigns for health checks, preventative health measures, pre- and post-natal screening, family planning, and immunization; (c) engagement of Roma in community health programmes; and (d) improved living conditions [8]. It placed emphasis on the well-being of women and children [8]. The persistent lack of accurate data on Roma health must first be addressed to inform healthcare service development [5, 15].

## Discussion

The NTRIS was the first national policy to explicitly include Roma [2, 20]. In 2019, the second review was completed by the EU and documented both the success and failures of its implementation [20]. Positive developments include continued success of the TRIP, formal acknowledgement of traveller ethnicity in 2017, inclusion of migrant groups in the Healthy Ireland Framework, and targeted Roma initiatives as part of the National Intercultural Health Strategy [20]. Other initiatives including Tusla, the child and family agency, are developing an initiative for retention of Roma and traveller children within the education system [20]. The EU also highlights the gap between planning and the implementation of Roma inclusion strategies [20]. Reasons for this are multifactorial, slow implementation of the NTRIS that has been linked to lack of and poor use of EU funds, poor participation by the Roma community, and negligible impact of Roma policies by the media [20]. An inequality gap between Roma and the majority population in Ireland is relevant for all 4 primary domains of the NTRIS: health, housing, education, and employment [20].

Review of the TRIP in 2016 demonstrated the advantages of actively engaging with the Roma for the provision of services, but also to provide Roma with a voice to communicate barriers to and issues in relation to service access and provision [15]. The Healthy Ireland Framework 2013–2025 includes migrant groups as part of an inclusive whole population approach to healthcare delivery, although does not refer to the Roma community specifically [15]. It has 4 primary objectives: increase the proportion of people healthy at all stages of life, reduce health inequalities, protect the population from threats to health and well-being, and create an environment where all individuals and sectors of society participate in achieving a healthy Ireland [14]. In addition to this, the HSE intercultural guide provides a framework for healthcare workers in provision of culturally appropriate care and will be continued as part of the second HSE National Intercultural Health Strategy (NIHS) 2018–2023 [1, 20].

Unlike the Healthy Ireland Framework, the NIHS refers directly to the health needs of the Roma population and other migrant groups [20]. The NIHS defines migrant as an umbrella term including Roma, refugees, asylum seekers, and undocumented migrants [20]. The primary goals of this most recent strategy include enhanced accessibility of services, address healthcare issues experienced by those from ethnic minority groups, provision of high quality culturally responsive services, build evidence base, and strengthen intercultural health [20]. Success of the first NIHS 2007–2012 included the HSE intercultural guide for staff, the emergency multilingual aid kit, peer health model for refugees and asylum seekers, and the Roma men’s training, development, and health literacy programme [20]. A major weakness of the first NIHS was poor data collection on ethnicity and failed implementation of ethnic equality monitoring limiting the analysis of projects on the ground [20].

Housing is particularly impacted due to the nationwide homeless and housing crisis [21]. The NTRIS described six actions in relation to housing for travellers, none referring to the Roma community [21]. According to the NRNA, 93% of anti-Roma discrimination occurred in relation to housing access [5, 21]. Inadequate housing and poor health status are linked with lower levels of education and unemployment [21]. According to census 2016, the level of unemployment for travellers is six times the majority population; there is no data available for Roma [2, 21]. As discussed for Roma, access to social welfare and employment support services is limited by the European Directive 2004/38 on freedom of movement and the habitual residence condition [21].

In the most recent Pandemic of Sars-CoV-2, Roma have featured as high-risk due to existing health and housing issues with increased vulnerability to the virus [22, 23]. Difficulties with self-isolation, physical distancing, and inadequate hygiene facilities in substandard accommodation put Roma at increased risk of contracting the virus [22]. Statistics from the European Commission states that up to 30% of Roma in Europe have no running water in their accommodation [24]. The higher rates of infectious disease, non-communicable chronic disease, and poor access to health services place Roma in a high-risk category for severe disease, hospitalisation, and death [5, 21–23]. Exclusion from social welfare support experienced by Roma was also exacerbated during covid [22]. A 2020 report by the department of Children and Youth Affairs stated that 18% of Roma households had greater than five children [22]. Educational disadvantage was exacerbated during the crisis due to low parental education level versus majority population, poverty, and lack of access to laptops and Internet for online teaching [22]. For some Roma children within the EU, closure of school meant loss of free daily meals [22]. Due to this, additional EU funding was set aside for disadvantaged children including Roma to access resources for online learning [22].

In March 2020, the Department of Health Ireland stated that both traveller and Roma communities would be treated as priority groups for diagnostic testing [22]. However, there were discrepancies between data collected from Pavee Point and the state [22]. Pavee Point documented sixty-one cases of covid amongst Roma with six deaths by March 2020; however, the HSE reported twenty-two cases of the virus with four deaths in the same period [22]. The data from Pavee Point suggests a 10% mortality rate in the Roma population which far exceeds that of the majority population in Ireland [22].

Across Europe, Roma are considered a high-risk priority group for Sars-CoV-2 [22, 23]. In Slovakia where 10% of the population is Roma, covid positivity rates have been up to 60%, and 22% of Roma who died from covid were less than 50 years [23]. In relation to covid vaccination status, the Roma have had low uptake in Europe [23]. Some reports state that vaccination uptake has been as low as 9% in Hungary and 1% in Slovakia for Roma communities [23]. Multiple explanations have been documented including misinformation, mistrust of healthcare systems, and poor healthcare access [23]. Positive initiatives by EU member states include the use of media campaigns, e.g. “Koronameterel” (Corona will die) in Slovakia [24]. Amalipe Roma NGO in Bulgaria ran a campaign for donation of old computers to Roma school children to access online learning [24].

Prior to covid 19, “MigRom” was a project run by the European Commission [25]. It investigated the experiences of Roma who migrated from Romania to Western Europe including Italy, France, Spain, and the UK [25]. The method included three surveys on ethnography, interviews through Romani language with Roma communities, and active involvement of Roma in research to promote Roma participation [25]. From 1989 on, there was an increase in migration of Roma from Romania due to border opening and dissolution of collective farms and state industry [25]. Romanian Roma migrants are a young age cohort [25]. As discussed previously, they have higher pregnancy rates and lower life expectancy than the majority population in many European countries, with approximately 80% less than 35 years of age [25]. Stable accommodation is key to social inclusion [25]. In the UK and Spain where Roma had access to long-term housing, there were improved school attendance and employment rates [25]. Traditional high rates of teenage pregnancy decreased, and demographic transition was seen through later pregnancies and purposeful postponement of future pregnancy [25]. Of note, many Irish Roma identify as Pentecostal religion which discourages contraception [1, 25].

Apart from the Irish NRIS, other European member states such as the UK attempted to implement strategies of Roma inclusion [25]. In Manchester city, proposals were put forward for education pathways and school admission protocols targeting the Roma; however, none were implemented [25]. A 3-year community outreach programme was also run in Manchester; it involved Roma peer led advice and support in collaboration with Manchester city council [25]. Roma Voices of Manchester is an NGO set-up by the Roma community [25]. In contrast, regions of France and Italy have local policies of Roma seclusion in attempts to remove Roma from cities such as Paris and Milan [25].

In an Irish context, the TRIP, although confined to the Tallaght region, has been the most successful example of collaboration between a healthcare service specific to Roma and mainstream healthcare [15]. The TRIP has demonstrated that initiatives like this are possible with Roma participation and trust building between relevant parties [15]. This project is applicable to a range of healthcare services in Ireland, promoting accessibility and cultural awareness [15]. Other successful local projects providing support to Roma include Waterford integration and support unit established in 2013 [5]. The Waterford service provides Roma health advocates for hospital appointments and additional support including applying for habitual residence [5]. Musicantia centre in Balbriggan established for Roma children and young people provides interpreters, advocacy, and a music school [5, 7]. These are examples of the positive actions of local organisations for the Roma community, but despite this, there is still a dearth of literature within the Irish healthcare context. This paper is intended as an overview of the Roma in Ireland. It is hoped that this paper will prove useful to Irish healthcare providers when navigating Roma culture in the provision of optimum and culturally competent care for a Roma child and their family.

## Data Availability

Not applicable.
